# Predictive Value of Preoperative Multidetector-Row Computed Tomography for Axillary Lymph Nodes Metastasis in Patients With Breast Cancer

**DOI:** 10.3389/fonc.2018.00666

**Published:** 2019-01-08

**Authors:** Chun-Fa Chen, Yu-Ling Zhang, Ze-Long Cai, Shu-Ming Sun, Xiao-Feng Lu, Hao-Yu Lin, Wei-Quan Liang, Ming-Heng Yuan, De Zeng

**Affiliations:** ^1^Department of Thyroid and Breast Surgery, The First Affiliated Hospital of Shantou University Medical College, Shantou, China; ^2^Department of Information, Cancer Hospital of Shantou University Medical College, Shantou, China; ^3^Department of Medical Imaging, The First Affiliated Hospital of Shantou University Medical College, Shantou, China; ^4^Cancer Research Center, Shantou University Medical College, Shantou, China; ^5^Department of Medical Oncology, Cancer Hospital of Shantou University Medical College, Shantou, China

**Keywords:** breast cancer, axillary lymph node metastasis, multidetector-row computed tomography, prediction, cortical thickness

## Abstract

**Introduction:** Axillary lymph nodes (ALN) status is an essential component in tumor staging and treatment planning for patients with breast cancer. The aim of present study was to evaluate the predictive value of preoperative multidetector-row computed tomography (MDCT) for ALN metastasis in breast cancer patients.

**Methods:** A total of 148 cases underwent preoperative MDCT examination and ALN surgery were eligible for the study. Logistic regression analysis of MDCT variates was used to estimate independent predictive factors for ALN metastasis. The prediction of ALN metastasis was determined with MDCT variates through receiver operating characteristic (ROC) analysis.

**Results:** Among the 148 cases, 61 (41.2%) cases had ALN metastasis. The cortical thickness in metastatic ALN was significantly thicker than that in non-metastatic ALN (7.5 ± 5.0 mm vs. 2.6 ± 2.8 mm, *P* < 0.001). Multi-logistic regression analysis indicated that cortical thickness of >3 mm (OR: 12.32, 95% CI: 4.50–33.75, *P* < 0.001) and non-fatty hilum (OR: 5.38, 95% CI: 1.51–19.19, *P* = 0.009) were independent predictors for ALN metastasis. The sensitivity, specificity and AUC of MDCT for ALN metastasis prediction based on combined-variated analysis were 85.3%, 87.4%, and 0.893 (95% CI: 0.832–0.938, *P* < 0.001), respectively.

**Conclusions:** Cortical thickness (>3 mm) and non-fatty hilum of MDCT were independent predictors for ALN metastasis. MDCT is a potent imaging tool for predicting ALN metastasis in breast cancer. Future prospective study on the value of contrast enhanced MDCT in preoperative ALN evaluation is warranted.

## Introduction

Axillary lymph nodes (ALN) status is a critical prognostic factor and has significant impact on tumor staging and treatment planning in patients with breast cancer (1, 2). Currently, axillary lymph node dissection (ALND) continues to be a primary procedure for nodal metastasis in breast cancer, which might increase the risk of developing short-term, and long-term complications, such as pain, numbness, impaired shoulder mobility, and lymphedema (3). Sentinel lymph node biopsy (SLNB) serves as an optimal choice for those without clinical or radiological evidence of ALN metastasis. It has been considered to be an ideal procedure for patients whose sentinel lymph node (SLN) are negative (4, 5) or those with 1–2 positive SLN (6, 7). However, second procedure of ALND or radiotherapy is unavoidable to adequately deal with axilla if SLN metastasis was found (8). Therefore, finding a reliable imaging modality to identify ALN metastasis preoperatively becomes particularly important.

Mammography, ultrasonography, and magnetic resonance imaging (MRI) are conventional imaging techniques for breast lesion evaluation, as well as for ALN status assessment (9). However, mammography is not a reliable modality for evaluating ALN status, primarily due to the difficulty in fully exposure of the whole axilla (10). Combination of ultrasonography with mammography was found to improve the sensitivity of metastatic nodes detection, however, the accuracy of ALN status evaluation with ultrasonography is mainly dependent on the operator's experience (11). Recently, the value of MRI in the evaluation lymph node status has been increasingly recognized. Whereas, due to time-consuming procedure and high cost of the examination, it remains not widely used in clinical practice (12, 13).

Multidetector-row computed tomography (MDCT) has emerged as a novel imaging technique in recent years. It is increasingly accepted by clinician to preoperatively assess regional lymph node status in a variety of cancers (14–16). MDCT can obtain high-quality multiplanar images in fast scan time and allow three-dimensional reconstruction (17). Furthermore, preoperative MDCT can be performed with the patient in the supine position, which facilitates simultaneous localization of the lesion, and evaluation of its extent, as well as examination of the skin, chest wall, and regional lymph nodes, including both axillae, internal mammary, and supraclavicular chains (5, 8, 18). However, preoperative MDCT for evaluating ALN status in patients with breast cancer remains under-investigated (18, 19). The aim of present study was to evaluate the predictive value of MDCT for ALN metastasis in patients with breast cancer.

## Materials and Methods

### Patients Enrollment

Patients with primary breast cancer had undergone MDCT and subsequent surgery from June 2016 to December 2017 at the Department of Thyroid and Breast Surgery in the first affiliated hospital of Shantou University Medical College were enrolled. The exclusion criteria included the following conditions: (1) Patients were diagnosed with distant metastasis; (2) Patients received neoadjuvant chemotherapy. All the patients received surgery for axillary staging, included SLNB, ALND or both. Information of patients' clinicopathological characteristic were collected. The number of lymph nodes resected were recorded, with subsequent pathological examination and confirmation by two pathologists. Informed consent for MDCT examination was obtained from each patient. The study was approved by the institutional review board.

### MDCT Protocol

All patients underwent examination with a 64-detector MDCT scanner (LightSpeed VCT 64, GE Healthcare, Milwaukee, WI, USA), with the following parameters: voltage of 120 kV, automatic tube current modulation (50–70 mAs), 1.75 mm pitch, 0.625 mm slice thickness, table speed of 175 mm/s, 2.5 rotation/s, and a matrix size of 512 × 512. All the MDCT examinations were performed on the patients from the level of the lower neck to the bottom of the thorax in the supine position with bilateral arms up over their heads. Single breath hold was requested during scanning in every case. Axial images were reconstructed at 5 mm interval in the coronal and sagittal planes. All three sets of images (axial, coronal, and sagittal) were evaluated using a picture archiving and communicating system. The mediastinal window settings consisted of a window level range 35–40, and a window width of 400.

### MDCT Evaluation of the Nodal Status

Images were reviewed on picture archiving and communicating system. Two radiologists with more than 10 years of experience in examining all the MDCT images without knowledge of pathologic results. The largest lymph node of affected side was measured on the MDCT image. The recorded parameters were included: (1) Long-axis diameter; (2) Short-axis diameter; (3) The ratio of long-/short-axis; (4) Cortical thickness; (5) Shape (oval or round); (6) Whether presence of fatty hilum.

### Statistical Analysis

Statistical analysis was performed by using SPSS statistical software (version 18.0, SPSS Inc., Chicago, IL, USA) and MedCalc Statistical Software (version 15.8, MedCalc Software bvba, Ostend, Belgium). Continuous variable was analyzed with Mann Whitney *U*-test, and categorical variable was compared with χ^2^-test using SPSS. Logistic regression analysis was performed to evaluate the variates associated with ALN metastasis, then those significant factors or marginal significant factor were determined with multivariate logistic regression analysis using an enter selection procedure. Receiver operating characteristic (ROC) curve analysis was performed using MedCalc to evaluate the MDCT variates of lymph node for diagnosing metastasis. The area under the ROC curve (AUC) was evaluated for diagnostic ability. The optimal cutoff value was based on the ROC curve with Youden's J statistic (J), J = sensitivity + specificity – 1. *P* < 0.05 was considered statistically significant.

## Results

### Patients Characteristic

**Figure 1:**
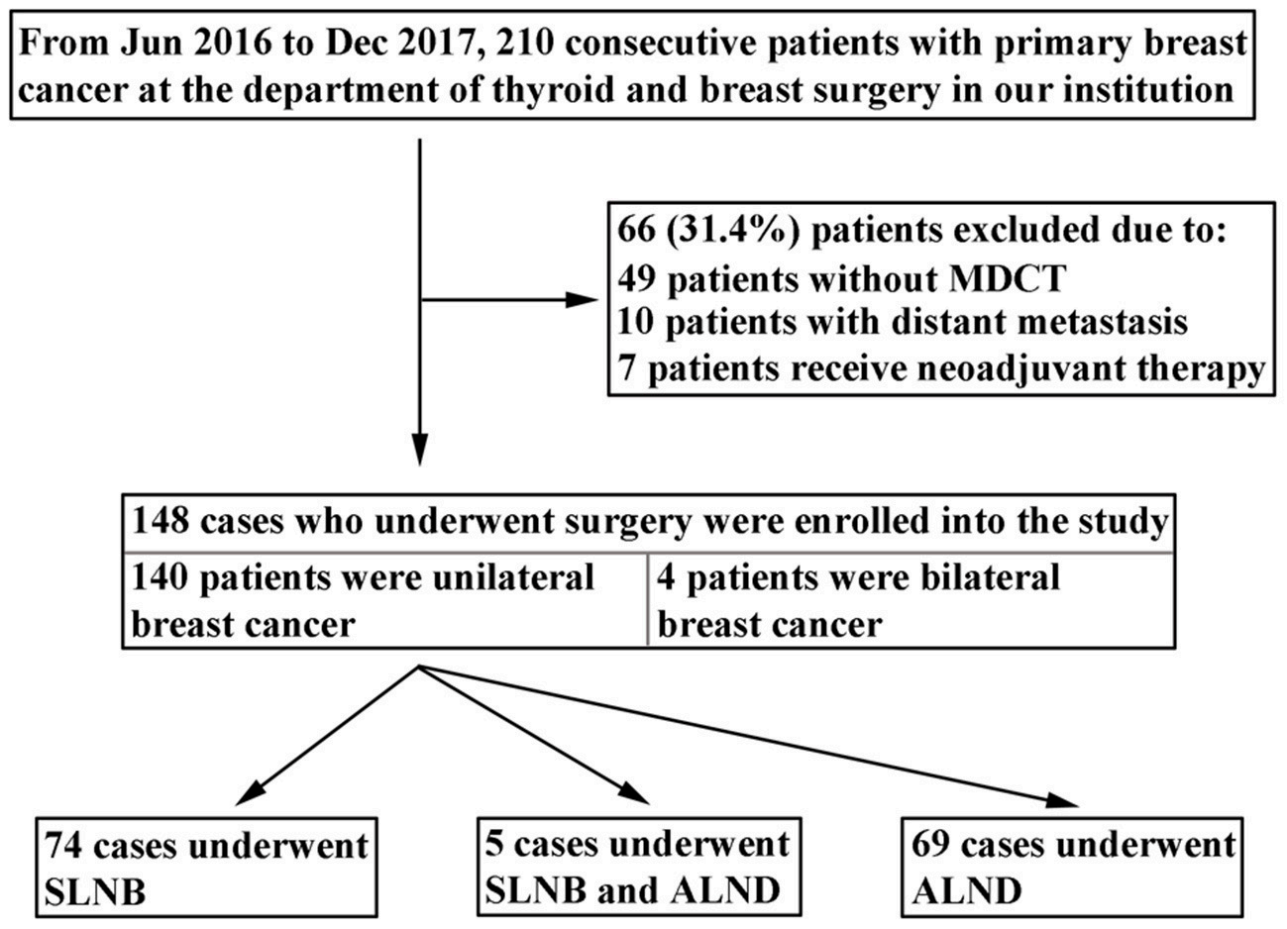
Flowchart of selecting patients with breast cancer.

A total of 210 consecutive patients with primary breast cancer hospitalized at the Department of Thyroid and Breast Surgery in the first affiliated hospital of Shantou University Medical College between June 2016 and December 2017 were initially reviewed for the study. All the patients underwent MDCT examination before surgery. Patients were excluded according to following conditions: (1) 49 patients had not undergone MDCT examination; (2) 10 patients presented with distant metastasis; (3) 7 patients had received neoadjuvant chemotherapy. Finally, 144 patients met the inclusion criteria and were recruited in the study. Of the 144 patients, 140 patients were diagnosed with unilateral breast cancer, and 4 patients with bilateral breast cancer. In the present study, both sides of ALN status were examined and evaluated with MDCT for patients with bilateral breast cancer. Therefore, there were 148 cases that underwent axillary surgery eligible for the analysis, including 74 cases underwent SLNB, 5 cases underwent both SLNB and ALND, and 69 cases underwent ALND (Figure 1).

All patients were females, with a median age of 52 years (range: 29–86 years). Tumor size was based on post-operative measurement of the tumor. The median tumor size of the 148 cases was 25 mm (range: 4–110 mm), including 40 (27.0%) cases were not more than 20 mm, 95 (64.2%) cases were larger than 20 mm but not <50 mm, and 13 (8.8%) cases were larger than 50 mm, respectively. According to the American Joint Committee on Cancer (AJCC) 7 staging system, 8 (5.4%) cases were stage 0, 22 (14.9%) cases were stage I, 86 (58.1%) were stage II, and 32 (21.6%) were stage III, respectively. Pathological review demonstrated that 140 (94.6%) cases were invasive breast carcinoma and 8 (5.4%) cases were ductal carcinoma *in situ* (DCIS). Among the 148 cases, 61 (41.2%) of them had ALN metastasis. Totally, 1,490 lymph nodes were dissected in all the cases, with a median of 5 (range: 1–35) lymph nodes dissected for each case. There were 1,072 lymph nodes dissected in the clinically suspected nodal involvement cases. Among them 310 (28.9%) were pathologically confirmed metastasis, with a median of 3 (range: 1–29) lymph nodes dissected for each case (Table 1).

**Table 1:** Patient characteristics.

**Characteristic**	**Median**	**No. of patients/ALN (%)**
Female		144 (100.0)
Age (years)	52 (29–86)	
Tumor size (mm)	25 (4–110)	
T ≤ 20		40 (27.0)
20 < T ≤ 50		95 (64.2)
T > 50		13 (8.8)
**STAGE**		
0		8 (5.4)
I		22 (14.9)
II		86 (58.1)
III		32 (21.6)
**HISTOLOGY**		
Invasive breast carcinoma		140 (94.6)
DCIS		8 (5.4)
ALN metastasis		61 (41.2)
No. of dissected ALN	5 (1–35)	1,490
No. of metastatic ALN	3 (1–29)	310/1,072 (28.9)

*ALN, axillary lymph nodes; DCIS, ductal carcinoma in situ*.

### MDCT Variables of Axillary Lymph Nodes

**Figure 2:**
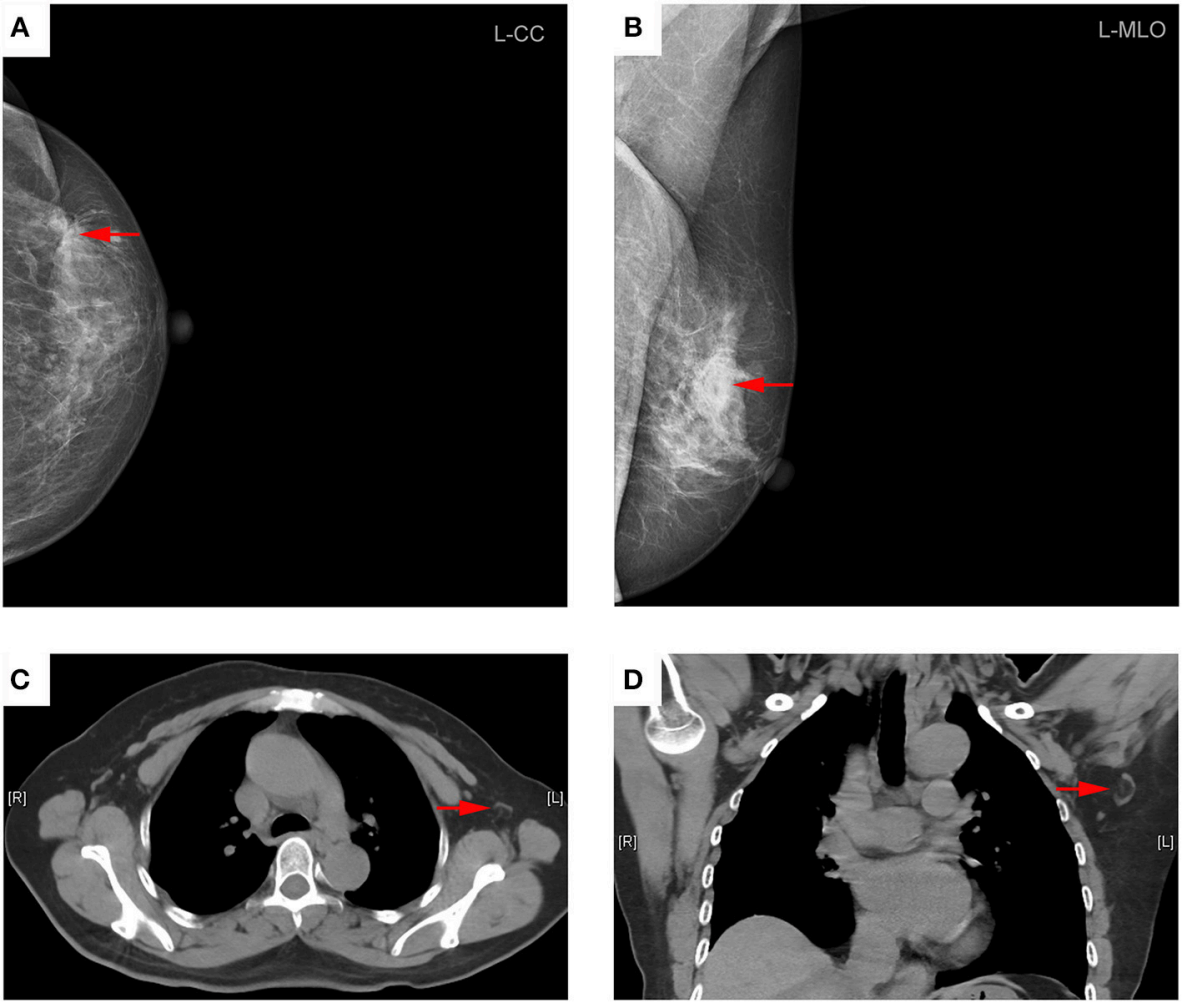
Representative mammography and computed tomography (CT) images of a 60-year-old woman with left primary breast cancer (stage pTisN0, DCIS). **(A)** mammography of craniocaudal image showed a mass in the lateral of the left breast, irregular, and slightly high-density shadows (red arrows). **(B)** Mammography of mediolateral oblique images showed a mass in the upper quadrant of the left breast, irregular, and slightly high-density shadows, about 21 × 13 mm in size, with rough edges and spicule sign (red arrows). **(C)** Transverse CT image showed an axillary lymph node with oval shape and presence of fatty hilum, a cortical thickness about 2 mm (red arrows). **(D)** Coronal CT image showed this lymph node with oval shape and presence of fatty hilum, a long-axis diameter of about 18 mm, and a short-axis diameter of about 8 mm (red arrows). The patient underwent sentinel lymph node biopsy, five lymph nodes were removed and proved to be pathologic negative.

**Figure 3:**
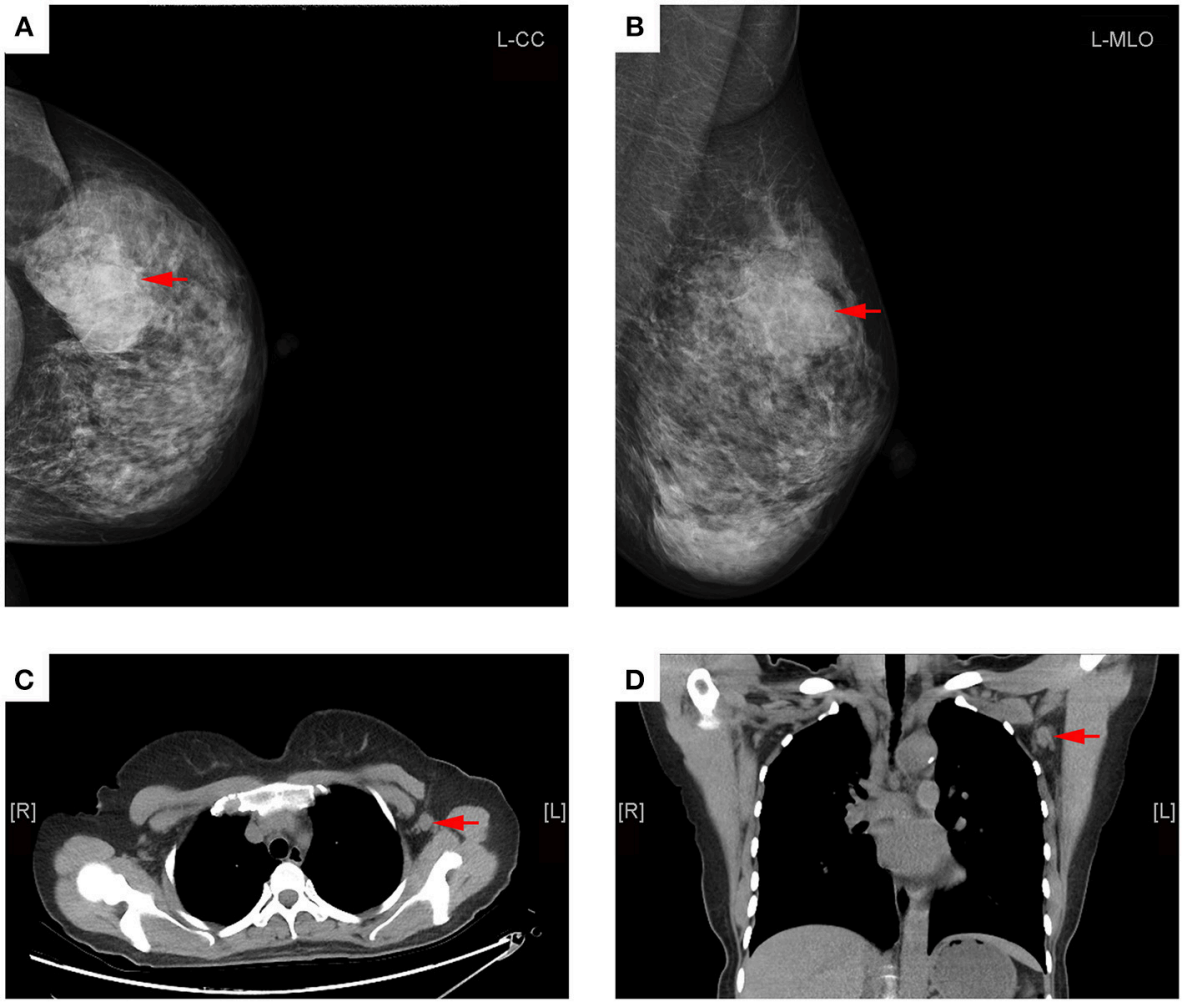
Representative mammography and MDCT images of a 45-year-old woman with left primary breast cancer (stage pT2N1). **(A)** Mammography of craniocaudal image showed a mass in the lateral of the left breast, lobulated, and slightly high-density shadows, about 56 × 36 mm in size, with rough edges and part of the edge was fuzzy (red arrows). **(B)** Mammography of mediolateral oblique images showed a mass in the upper quadrant of the left breast, lobulated, and slightly high-density shadows (red arrows). **(C)** Transverse CT image showed an axillary lymph node with round shape and non-fatty hilum, a cortical thickness about 11 mm (red arrow). **(D)** Coronal CT image showed this lymph node with round shape and no fatty hilum, a long-axis diameter of about 15 mm, and a short-axis diameter of about 13 mm (red arrows). The patient underwent axillary lymph node dissection, 15 lymph nodes were removed, and two lymph nodes were proved to be pathologic positive.

As showed in Table 2, the average short-axis diameter of metastatic ALN was significantly longer than that of non-metastatic ALN (10.0 ± 4.3 mm vs. 7.4 ± 2.5 mm, *P* < 0.001). The average ratio of long-/short-axis of metastatic ALN was significantly shorter than that of non-metastatic ALN (1.5 ± 0.4 vs. 1.8 ± 0.6, *P* = 0.003). The thickness of cortex in metastatic ALN was significantly thicker than that in non-metastatic ALN (7.5 ± 5.0 mm vs. 2.6 ± 2.8 mm, *P* < 0.001). Compared with metastatic ALN, more non-metastatic ALN presented with an oval shape (*P* < 0.001) and a fatty hilum (*P* < 0.001) (Figures 2, 3). No significant difference was found in long-axis diameter between metastatic ALN and non-metastatic ALN (14.7 ± 6.4 mm vs. 12.5 ± 4.2 mm, *P* = 0.065) (Table 2).

**Table 2:** Comparison of ALN metastasis with MDCT variables.

**Characteristic**	**All patients**	**ALN metastasis**		***P*-value**
		**Negative (*n* = 87)**	**Positive (*n* = 61)**	
Long-axis diameter (mm)	13.4 ± 5.3	12.5 ± 4.2	14.7 ± 6.4	0.065
Short-axis diameter (mm)	8.5 ± 3.6	7.4 ± 2.5	10.0 ± 4.3	<0.001
Ratio of long-/short-axis	1.7 ± 0.5	1.8 ± 0.6	1.5 ± 0.4	0.006
Cortical thickness (mm)	4.6 ± 4.5	2.6 ± 2.8	7.5 ± 5.0	<0.001
Shape (%)				<0.001
Oval	75 (50.7)	59 (78.7)	16 (21.3)	
Round	73 (49.3)	28 (38.4)	45 (61.6)	
Fatty hilum (%)				<0.001
Yes	68 (45.9)	61 (89.7)	7 (10.3)	
No	80 (54.1)	26 (32.5)	54 (67.5)	

*ALN, axillary lymph nodes; MDCT, multidetector-row computed tomography*.

### Univariate and Multivariate Analysis

Univariate analysis showed that short-axis diameter (>9 mm), ratio of long-/short-axis (<1.7), cortical thickness (>3 mm), oval or round shape, and non-fatty hilum were significantly associated with ALN metastasis, except for long-axis diameter (>14 mm). Multivariate analysis demonstrated that cortical thickness (>3 mm) and non-fatty hilum were independent predictors for ALN metastasis (OR: 12.32, 95%, CI: 4.50–33.75, *P* < 0.001; OR 5.38, 95% CI: 1.51–19.19, *P* = 0.009) (Table 3).

**Table 3:** Univariate and multivariate logistic regression analysis for ALN metastasis.

**Variable**	**Univariable analysis**		**Multivariable analysis**	
	**OR (95% CI)**	***P*-value**	**OR (95% CI)**	***P*-value**
**LONG-AXIS DIAMETER**				
≤14.0 mm	1		1	
>14.0 mm	1.95 (0.98–3.90)	0.058	1.39 (0.39–4.96)	0.617
**SHORT-AXIS DIAMETER**				
≤9 mm	1		1	
>9 mm	4.65 (2.20–9.83)	<0.001	0.93 (0.25–3.42)	0.916
**RATIO OF LONG-/SHORT-AXIS**				
≥1.7	1		1	
<1.7	3.50 (1.73–7.06)	<0.001	1.92 (0.59–6.20)	0.279
**CORTICAL THICKNESS**				
≤3 mm	1		1	
>3 mm	27.73 (11.28–68.22)	<0.001	12.32 (4.50–33.75)	<0.001
**SHAPE**				
Oval	1		1	
Round	5.93 (2.87–12.25)	<0.001	0.86 (0.24–3.08)	0.814
**FATTY HILUM**				
Yes	1		1	
No	18.10 (7.28–45.03)	<0.001	5.38 (1.51–19.19)	0.009

*ALN, axillary lymph nodes; OR, odds ratio; CI, confidence interval*.

### Comparison of Axillary Lymph Nodes Metastasis Prediction Performance

**Figure 4:**
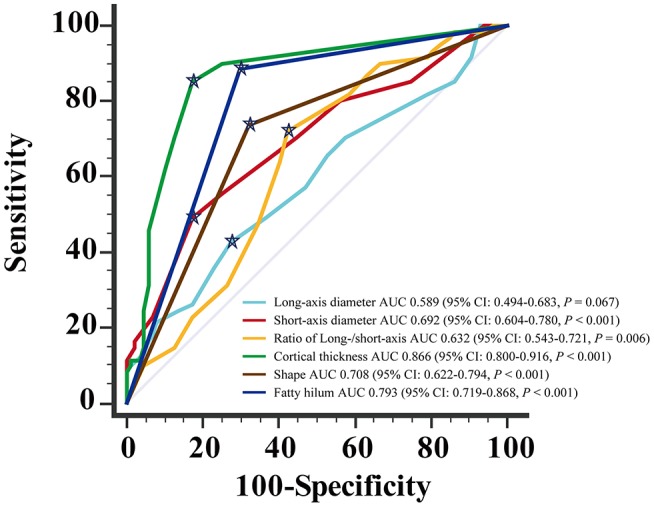
Receiving operating characteristic (ROC) curve analysis for each of MDCT parameter. 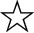 Showed the optimal cut-off.

**Figure 5:**
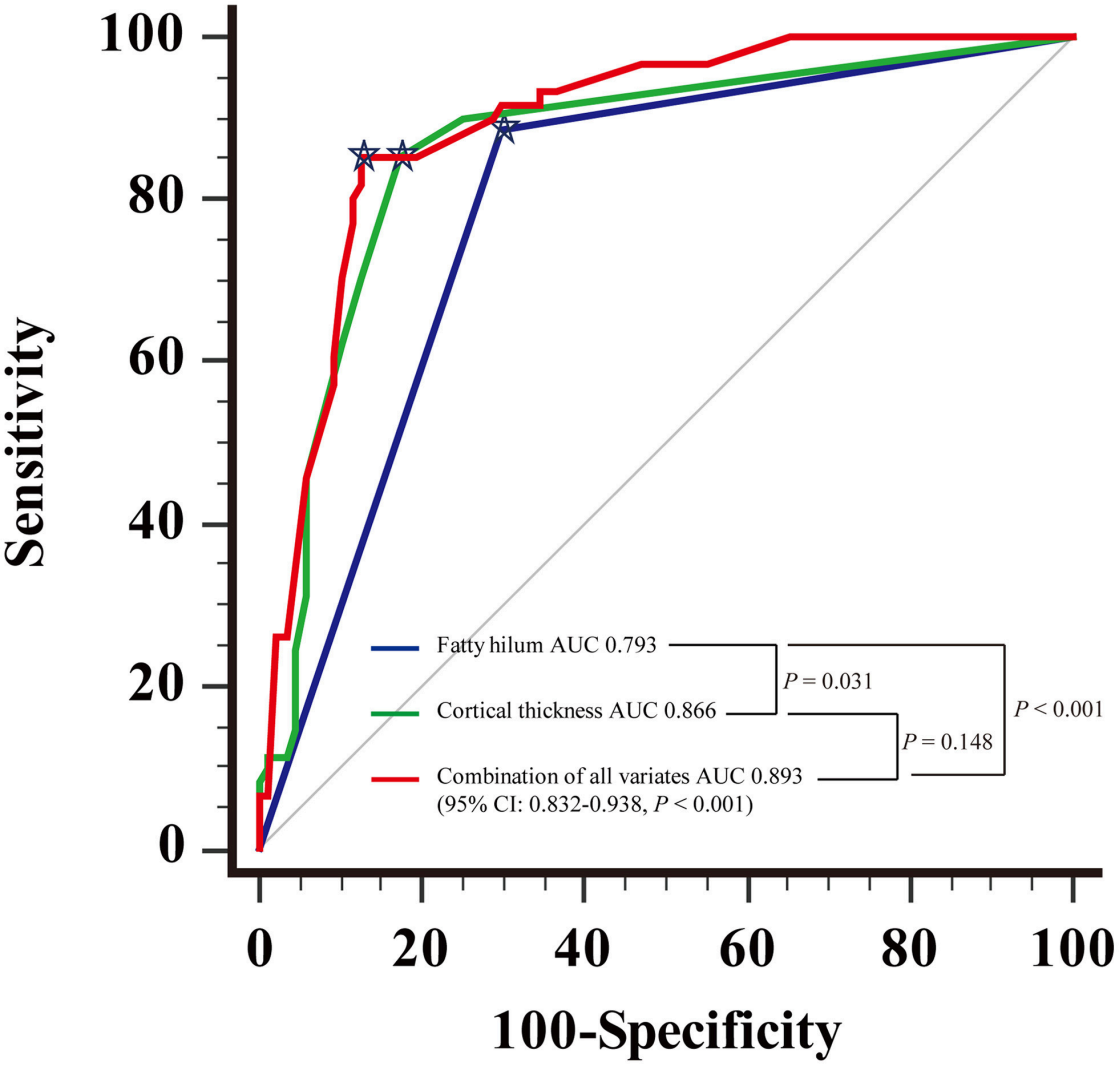
Receiving operating characteristic (ROC) curve analysis showed combination of all the imaging parameters and comparison of Areas under the curve (AUC). 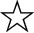 Showed the optimal cutoff.

For the diagnosis of metastatic ALN as showed in Table 4, the ROC based on multivariate analysis showed that 14.5 mm was the optimal cutoff value of long-axis diameter, with an AUC of 0.589 (95% CI: 0.494–0.683, *P* = 0.067). The sensitivity, specificity and J were 42.6, 72.4, and 15.0%, respectively. The optimal cutoff value of short-axis diameter was 9.5 mm, with an AUC of 0.692 (95% CI: 0.604–0.780, *P* < 0.001). The sensitivity, specificity and J were 49.2, 82.8, and 31.9%, respectively. The optimal cutoff value of ratio of long-/short-axis was 1.7, with an AUC of 0.632 (95% CI: 0.543–0.721, *P* = 0.006). The sensitivity, specificity and J were 72.1, 57.5, and 29.6%, respectively. The optimal cutoff value of cortical thickness was 3 mm, with an AUC of 0.866 (95% CI: 0.800–0.916, *P* < 0.001), and the sensitivity, specificity, and J were 85.3, 82.8, and 68.0%, respectively. The AUC of shape of ALN was 0.708 (95% CI: 0.622–0.794, *P* < 0.001), and the sensitivity, specificity, and J were 73.8, 67.8, and 41.6%, respectively. The AUC of fatty hilum of ALN was 0.793 (95% CI: 0.719–0.868, *P* < 0.001), and the sensitivity, specificity, and J were 88.5, 71.1, and 58.6%, respectively (Figure 4). Combined-analysis with all imaging variates indicated that the AUC was 0.893 (95% CI: 0.832–0.938, *P* < 0.001), and the corresponding sensitivity, specificity, and J were 85.3, 87.4, and 72.6%, respectively. As mentioned above, cortical thickness and non-fatty hilum were independent predictive factors for ALN metastasis. In addition, cortical thickness showed a significant improvement in prediction of ALN metastasis than non-fatty hilum (*P* = 0.031). Combined-analysis with all imaging variates also showed a significant improvement in the prediction of ALN metastasis than non-fatty hilum (*P* < 0.001), however, no significant difference was found when compared with cortical thickness (*P* = 0.148) (Figure 5).

**Table 4:** Optimal cut-off values for diagnosis of ALN metastasis with MDCT.

**MDCT variates**	**Optimal cut-off value**	**Sensitivity (%)**	**Specificity (%)**	**Youden index (%)**
Long-axis diameter	14.5 mm	42.6	72.4	15.0
Short-axis diameter	9.5 mm	49.2	82.8	31.9
Ratio of long-/short-axis	1.7	72.1	57.5	29.6
Cortical thickness	3 mm	85.3	82.8	68.0
Shape		73.8	67.8	41.6
Fatty hilum		88.5	71.1	58.6
Combination of all variates		85.3	87.4	72.6

*ALN, axillary lymph nodes; MDCT, multidetector-row computed tomography*.

## Discussion

Accurate evaluation of the axilla is a crucial component in preoperative staging and tailoring therapy for patients with primary breast cancer (2, 20). Conventionally, mammography and ultrasonography are routine imaging procedures for breast cancer diagnosis, as well as evaluation of ALN status (21, 22). While breast MRI is frequently performed to assess the extent of lesion involvement after a histological confirmation of malignancy (23). Despite advances in these modalities, limitations remain in terms of sensitivity, and specificity, as well as cost-effectiveness.

Currently, the National Comprehensive Cancer Network (NCCN) clinical practice guidelines (Breast Cancer) does not recommend routine chest CT imaging for patients with stage I-II breast cancer, while patients with stage III-IV disease typically need undergo CT or PET/CT imaging (24). However, even among early stage patients, CT is recommended if patients have pulmonary symptoms and, importantly, many patients will undergo chest CT for other reasons (e.g., chest pain). Thus, lung nodules may be incidentally detected even in early stage breast cancer patients, and these nodules could be regarded as suspicious of malignancy. In clinical practice in our institution, we routinely performed contrast CT or PET-CT in patients with stage III-IV breast cancer. However, for patients with stage I-II, we usually preferred plain chest CT scan. If any abnormal lesion is found, then, we will use the contrast CT scan. The purpose of this plain CT scan is to find out whether there is nodule in the lung, which is in line with the American Thoracic Society and American College of Chest Physicians recommendation of using low dose CT screening for pulmonary nodules (25). MDCT scanners are capable of acquiring multiple CT datasets in each rotation of the X-ray tube. They can scan large anatomic areas several times faster than single-detector helical CT scanners (18, 26). Moreover, through thinner collimation, MDCT markedly improves the spatial and time resolution of images acquired to detect lesions (18, 26, 27). Therefore, there is potential usefulness of MDCT as a method for accurate evaluation of ALN status. However, application of MDCT for the evaluation of ALN status is scarcely reported.

The present study demonstrated that short-axial diameter of metastatic ALN was significantly longer than that of non-metastatic ALN in MDCT scan. This result was in line with the response evaluation criteria in solid tumors 1.1, recommending short-axis diameter to distinguish benign from malignant lymph node (28). Moreover, average ratio of long-/short-axis diameter of metastatic ALN was significantly lower than that of non-metastatic ALN. Consistent results in previous studies of MDCT and MRI also suggested that short-axial diameter and ratio of long-/short-axis were crucial parameters in predicting lymph node metastasis (29–31).

At present, the optimal cutoff value of short-axis diameter for differentiating metastatic from normal lymph nodes remains undetermined. It has been reported that the optimal cutoff value of short-axis diameter could be ranged from 5.7 to 15 mm in distinct types of carcinomas (14–16, 28, 32–34). ROC analysis from our study suggested that 9.5 mm was an optimal cutoff value of short-axis diameter for distinguishing nodal metastasis. This proposed cutoff value was supported by but lower than that of March's finding in CT scan, which accepted 10 mm of short-axis for diagnosing lymph node metastasis in breast cancer (32). A recent study by Imai, N. and colleagues showed that, by CT scan, patients with ≥3 nodes meeting the criterion of both having a long-axis diameter ≥10 mm and a short-axis diameter ≥5 mm could be diagnosed with ALN metastasis (35). In our study, the average long-axis diameter in metastatic ALN was longer than that in non-metastatic counterparts by MDCT scan, however, no statistically significant difference was found between these two sets of ALN. In consideration of the size of ALN alone was not sufficient to determine the status of ALN. We, therefore, further evaluated the predictive values of the ratio of long-/short-axis and the morphology of ALN.

Usually, the ratio of long-/short-axis in normal or hyperplastic lymph nodes is larger than metastatic lymph nodes (14, 31, 36). Using MDCT, we found that ratio of long-/short-axis <1.7 was more likely to be a malignant nodal lesion. Study of high-resolution helical CT for evaluating small ALN in patients with breast cancer indicated that a malignant nodes could be determined when ratio of the longest axis to the shortest axis <2.0 (36). Another study by Li et al. assessing the imaging variates of bladder cancer patients, who had undergone radical cystectomy, demonstrated that the ratio of long-/short-axis <2.0 indicated metastases (14). The cutoff value in their results were given through a dichotomy of direct calculation, whereas, the optimal cutoff value was derived from a ROC analysis in our study, which is, methodologically and statistically, more appropriate and reliable.

Subject to internal structure of lymph node, breast cancer cells usually enter the subcapsular sinus of cortex through afferent lymphatic vessels, where they eventually situate and proliferate. Hence, the cortex of metastatic lymph node is often found thickening in radiological imaging (37, 38). Generally, cortical thickness of <3 mm is considered to be a normal lymph node, but thresholds may be different among various institutions with distinct experiences (36, 38–40). The present study showed that the cortex of metastatic ALN was significantly thicker than that of non-metastatic ALN in MDCT scan. In addition, we demonstrated that cortex >3 mm was a strong indicator for predicting ALN metastasis, with an AUC of 0.866. Uematsu et al. found that 90% of metastatic lymph nodes accompany of concentric cortex larger than 2 mm (36). However, in these studies, most patients were in early stages (including stage I or II), while patients in our study comprised of those with stage I to III diseases. Therefore, the cutoff value of cortex in present result might be more representative and credible.

Morphologically normal lymph nodes are typical oval with a fatty hilum. As metastases involving lymph nodes and progressing, the normal nodal tissue was replaced by cancer cells, and they begin to lose their fatty hilum and tend to be round (41). Kutomi et al. using multivariate analysis revealed that rounded lymph nodes with non-fatty hilum in MDCT imaging was an independent predictor of ALN metastasis (42). However, in-depth analysis on the structure of hilum and cortex was not carried out in their study. Our study confirmed and supported their perspective that round shape and non-fatty hilum were significantly associated with metastatic lymph nodes. Additionally, we also found that presence of non-fatty hilum was superior to round shape in predicting ALN metastasis.

**Figure 6:**
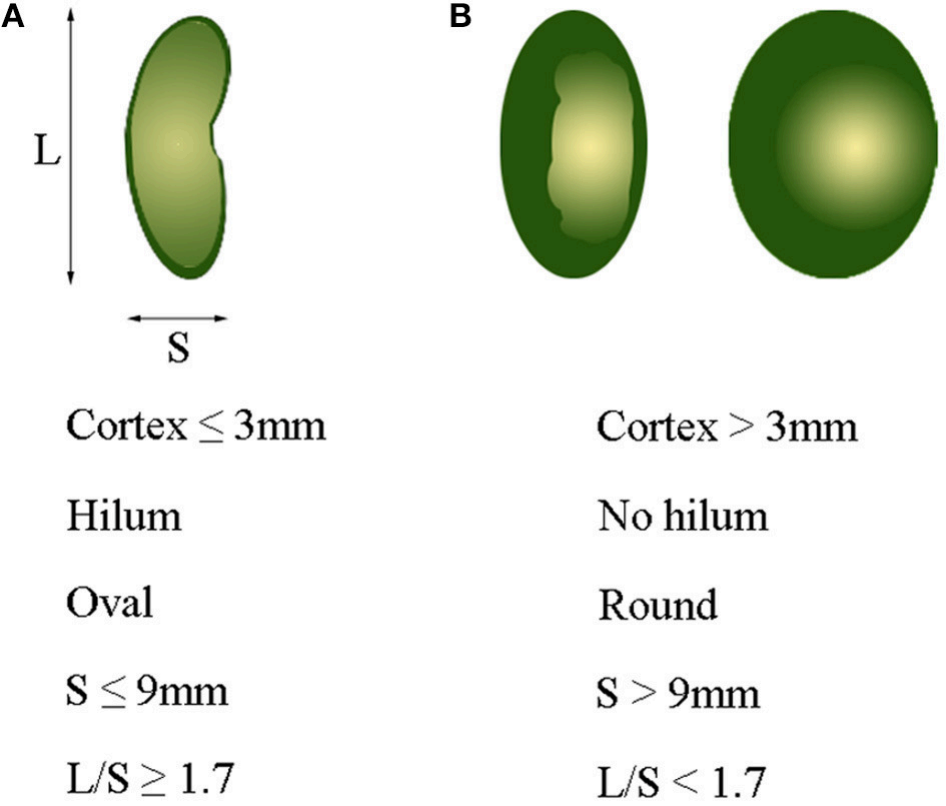
Optimal cutoff value of variables for identifying metastatic axillary lymph nodes. **(A)** Cortex ≤3 mm, presence of hilum, oval shape, S ≤ 9 mm, and L/S ≥ 1.7 usually indicate a normal lymph node. **(B)** Cortex >3 mm, non-hilum, round shape, S > 9 mm, and L/S < 1.7 mostly indicate a metastatic lymph node. L, long-axis diameter; S, short-axis diameter; L/S, ratio of long-/short-axis.

Axillary ultrasonography is often used as a screening tool for lymph node assessment, but cannot replace SLNB for axillary staging, primarily attributed to its limited sensitivity for minor axillary metastatic burden (43). Several studies reported that the sensitivity and specificity of ALN by CT examination ranged from 60 to 78%, and from 76 to 97%, respectively (36, 44, 45). In our study, the sensitivity of MDCT based on fatty hilum, cortical thickness and combined-variate analysis were 88.5, 85.3, and 85.3%, respectively, and the corresponding specificity were 71.1, 82.8, and 87.4%, respectively. It was also found that the accuracy of ALN evaluation based on combined-variate analysis was significantly better than single-variate analysis, based on either fatty hilum or cortical thickness. Therefore, combined-variate analysis is more appropriated and reliable when using MDCT to identify ALN metastases. To assess ALN with diffusion-weighted MRI in combination with routine and dynamic contrast MRI, Razek et al. reported that the accuracy of 95.6%, sensitivity of 93%, specificity of 100% and AUC of 0.974 (46). Combination of diffusion-weighted MRI with morphological and dynamic MRI findings is more accurate in differentiating metastatic- from benign-ALN, which is superior to our findings with MDCT. Wahl et al. reported that FDG-PET was 61% sensitive and 80% specific for axillary metastases, with a positive predictive value of 62% and a negative predictive value of 79% (47). The sensitivity and specificity in our study were higher than theirs, which may be attributed to the using of combined-analysis with all imaging variates of lymph nodes. In short, ALN with a greater number of parameters, including cortex ≤3 mm, presence of fatty hilum, oval shape, short axis (S) ≤9 mm, and long-/short-axis ratio (L/S) ≥1.7, are more likely to be negative nodes, as shown in Figure 6A, while ALN with a greater number of parameters, including cortex >3 mm, absence of fatty hilum, round shape, short axis (S) >9 mm, and long-/short-axis ratio (L/S) <1.7, are suspicious of being a metastatic lymph nodes, as shown in Figure 6B.

Through MDCT examination, we can preliminarily assess the lymph node status. If ALN on MDCT are more like to be negative, we will be more likely to choose SNLB. If MDCT shows apparent metastases of ALN, we will consider to perform ALND. In this study, if there is only one suspicious positive lymph node on MDCT imaging, and found that 1–2 positive sentinel nodes during surgery, and meet the inclusion criteria of Z0011 trial (7), further ALND may be avoided. We hope that future prospective clinical trials can validate this result, and provide evidence for triaging the patients either to receive SLNB or ALND. This study is helpful for preoperatively evaluating the status of ALN, particularly for those with typical hilar, and cortical structures, but it remains not able to replace SLNB. Although our study focused only on ALN, the detection of any distant metastases in these patients could alter treatment from neoadjuvant or surgical to palliative management. The drawbacks of the study are, firstly, the nature of a retrospective study and the relatively small sample size from a single institution, which might compromise the representativeness of the study. Secondly, there might be bias stems from the difficulty in conducting a paired-comparison between MDCT imaging and pathological result in each lymph node. Lastly, enhancement scanning was not carried out in this study, which may reduce the sensitivity of MDCT examination, and future study of MDCT with enhancement scanning is needed.

## Conclusion

In conclusion, cortical thickness and non-fatty hilum were independent predictive factors in MDCT for predicting ALN metastasis. The optimal cutoff value of cortical thickness in predicting ALN metastasis in patients with breast cancer was 3 mm. ALN with cortex >3 mm and non-fatty hilum was considered highly metastasis. MDCT is a potent imaging tool for predicting ALN metastasis in breast cancer. Prospective study on the value of contrast enhanced MDCT for preoperative ALN evaluation in patients with breast cancer is warranted.

## Author Contributions

C-FC and DZ conceived and designed the project. Z-LC analyzed the imaging. Y-LZ, W-QL, and M-HY collected the patients' characteristic data. C-FC and Y-LZ prepared the figures and tables. S-MS, X-FL, and DZ analyzed and interpreted the data. C-FC, H-YL, and DZ wrote the manuscript. DZ approved the final version to be submitted. C-FC and DZ are the corresponding authors. All the authors read and approved the final manuscript.

### Conflict of interest Statement

The authors declare that the research was conducted in the absence of any commercial or financial relationships that could be construed as a potential conflict of interest.

## Abbreviations

ALN: axillary lymph nodes
ALND: axillary lymph node dissection
AUC: area under the ROC curve
CI: confidence interval
CT: computed tomography
DCIS: ductal carcinoma *in situ*
J: Youden's J statistic
MDCT: multidetector-row computed tomography
MRI: magnetic resonance imaging
OR: odds ratio
ROC: receiver operating characteristic
SLN: sentinel lymph node
SLNB: sentinel lymph node biopsy.
